# Disruption of paired-associate learning in rat offspring perinatally exposed to dioxins

**DOI:** 10.1007/s00204-013-1161-y

**Published:** 2013-11-29

**Authors:** Masaki Kakeyama, Toshihiro Endo, Yan Zhang, Wataru Miyazaki, Chiharu Tohyama

**Affiliations:** 1 Laboratory of Environmental Health Sciences, Center for Disease Biology and Integrative Medicine, Graduate School of Medicine, The University of Tokyo, 7-3-1 Bunkyo-ku, Tokyo, 113-0033 Japan; 2 Department of Neurobiology and Behavior, Graduate School of Biomedical Sciences, Nagasaki University, 1-12-4 Sakamoto, Nagasaki, 852-8501 Japan; 3 Department of Public Health, Faculty of Life Sciences, Kumamoto University, 1-1-1 Honjyo, Kumamoto, 860-8556 Japan

**Keywords:** Behavior, Dioxin, Higher brain function, Neurotoxicity

## Abstract

The prevalence of cognitive abnormalities in children has partly been ascribed to environmental chemical exposure. Appropriate animal models and tools for evaluating higher brain function are required to examine this problem. A recently developed behavioral test in which rats learn six unique flavor-location pairs in a test arena was used to evaluate paired-associate learning, a hallmark of the higher cognitive function that is essential to language learning in humans. Pregnant Long-Evans rats were dosed by gavage with 2,3,7,8-tetrachlorodibenzo-*p*-dioxin (TCDD) or 2,3,7,8-tetrabromodibenzo-*p*-dioxin (TBDD) at a dose of  0, 200, or 800 ng/kg (referred as Control, TCDD-200, TCDD-800, TBDD-200, or TBDD-800, hereafter) on gestational day 15, and the offspring was tested during adulthood. Paired-associate learning was found to be impaired in the TCDD-200 and TBDD-200 groups, but not in either group exposed to 800 ng/kg, the observations of which were ensured by non-cued trials. As for the emotional aspect, during habituation, the TCDD-200 and TBDD-200 groups showed significantly longer latencies to enter the test arena from a start box than the Control, TCDD-800, and TBDD-800 groups, suggesting that the TCDD-200 and TBDD-200 groups manifested anxiety-like behavior. Thus, both the chlorinated dioxin and its brominated congener affected higher brain function to a similar extent in a nearly identical manner. Use of the behavioral test that can evaluate paired-associate learning in rats demonstrated that in utero and lactational exposure to not only TCDD but also TBDD perturbed higher brain function in rat offspring in a nonmonotonic manner.

**Electronic supplementary material:**

The online version of this article (doi:10.1007/s00204-013-1161-y) contains supplementary material, which is available to authorized users.

## Introduction

Developing fetuses and children are unusually susceptible to environmental hazards because of their unique growth and developmental processes and immature metabolic systems, physiology, and behavior (Wigle 2003; World Health Organization 2006). Although serious health problems due to devastating environmental pollution in local areas are less likely to occur in modern society because of the implementation of appropriate legislative measures, exposure to chemicals, including agrochemicals and environmental pollutants, has been reported to be common in vulnerable populations, such as infants (Grandjean and Landrigan 2006; Szpir 2006). Thus, it is important to note that chemical exposure at low doses during the perinatal period might disturb the developing brain and perturb higher brain function, particularly later in life (Thompson et al. 2009).

Behavioral alterations or learning impairments have often been used as representative endpoints of neurotoxicity. For risk assessment of chemicals, neurotoxicity guidelines have been proposed and implemented by the United States Environmental Protection Agency (US EPA 1998), with a significant revision by the Organisation for Economic Co-operation and Development (Organisation for Economic Co-operation and Development 2007). In these guidelines, a behavioral test battery has been proposed for examining developmental neurotoxicity in terms of spontaneous activity, learning and memory, and neuropathology, and the use of the battery has been validated by international collaborative activities. Thus, the use of these tests in regulatory science has been suggested by international, national, and private sectors. However, there have been some controversies on the test methods with respect to the sensitivity and specificity of the neurotoxicity tests, the kinds of endpoints that should be included, the complexity of the test protocol, and the large variability of some endpoints (Kuwagata 2012; Makris et al. 2009). The scientific literature used to derive the acceptable daily intake or tolerable daily intake values is limited. In addition, for the safety evaluation and/or risk assessment of chemicals, it is a long-lasting and yet unsolved problem how animal data can be extrapolated to human situations. Phenotypes of higher brain function as well as endpoints of neurotoxicity elicited by chemicals need to be carefully studied in laboratory animals in comparison with humans. In addition, there is a need to update the test battery in order to incorporate newly developed cognitive and behavioral tests. To this end, in this study, we applied a recently developed test that can evaluate paired-associate learning in rats (Tse et al. 2007) in a developmental neurotoxicological study of dioxins. Paired-associate learning is a form of learning that involves the pairing of two naturally unrelated items (e.g., a word and a number) in memory, which is essential for language learning and for developing a vast store of knowledge in humans. In a test of paired-associate learning, a subject is required to recall one member of a pair from the other one. The performance of paired-associate learning has been recognized as a highly sensitive marker for detecting even subtle signs of cognitive impairment in clinical practice (Lowndes and Savage 2007). In the protocol of this paired-associate learning test, rats can learn a putative restaurant map involving a set of six paired associations with the flavors of food and their spatial locations (Tse et al. 2007). This test requires not only the hippocampal region (Day et al. 2003) but also the prefrontal cortex (Tse et al. 2011). This higher brain function task is a novel approach that can be applied to investigate the mechanisms underlying the toxicity of chemicals in the developing brain as well as neurotoxicity safety and risk assessments across species.

As a model chemical, we used 2,3,7,8-tetrachlorodibenzo-*p*-dioxin (TCDD), which is the most toxic congener of dioxins. Dioxins are a group of chemicals that are inadvertently produced during combustion and many other industrial processes. Epidemiological studies on children cohorts in areas around Lake Michigan, USA, or in Holland have shown that higher brain function, as measured by IQ, cognitive tests, and preferences to boy’s or girl’s play, is affected by dioxins and polychlorinated biphenyls (Jacobson and Jacobson 1996, 2002; Patandin et al. 1999; Vreugdenhil et al. 2002a, b), the latter of which has congeners that are classified as dioxins because of their similar actions through the aryl hydrocarbon receptor. The exposure level was found to be too low to overtly affect the mothers’ health. In experimental studies, rodent offspring born to dams that were exposed to TCDD manifested behavioral abnormalities in a variety of tests, such as the T-maze visual reversal task (Schantz et al. 1996), operant conditioning (Hojo et al. 2002, 2008; Markowski et al. 2001, 2002), fear conditioning (Haijima et al. 2010; Mitsui et al. 2006), and an active avoidance task (Nishijo et al. 2007). Such a developmental neurotoxicity test battery confirmed the consistency of their overall test results. Intriguingly, the learning performance of TCDD-exposed offspring was affected in the schedule-controlled operant behavioral test when both fixed ratio (FR) and differential reinforcement of low rates of responding (DRL) tasks were used, whereas no effect was observed by either the FR or DRL task only (Hojo et al. 2008), suggesting that the developmental effects of TCDD exposure were not apparent in such a simple learning test. Thus, we hypothesized that the recently developed behavioral test comprising complex tasks for rats can be used to characterize the developmental neurotoxicity of dioxins.

Besides chlorinated dioxin congeners, the formation and release of brominated dioxin congeners into the environment and their possible health effects have been studied, although information in terms of the comparative toxicity of chlorinated and brominated compounds is limited. A most recent review on the risk assessments of brominated dioxin compounds (van den Berg et al. 2013) has proposed that a similar toxicity equivalent factor similar to the one for chlorinated congeners can be applied to brominated congeners based on a limited number of studies on the toxicity of brominated dioxin congeners. Thus, in the present study, we applied behavioral tests of paired-associate learning and studied the possible effects of TCDD or 2,3,7,8-tetrabromodibenzo-*p*-dioxin (TBDD) on the formation of paired-associate learning in rat offspring.

## Materials and methods

### Reagents and rodent diets

TCDD and TBDD (purity, 99.9 %) were purchased from Cambridge Isotope Laboratories (Andover, MA, USA). TCDD and TBDD solutions were prepared by diluting them with vehicle, which was 4.0 % n-nonane in corn oil (Sigma-Aldrich Japan, Osaka, Japan). Laboratory rodent chow (Lab MR Stock) was purchased from Nosan Corporation, Yokohama, Japan. Flavored rat chow pellets (190 mg each) used as reward were purchased from Bio-Serv (Frenchtown, NJ, USA). Other reagents were obtained from Nakalai Tesque, Inc. (Kyoto, Japan).

### Animals and exposure

Long-Evans hooded rats were purchased from the Institute for Animal Reproduction (Ibaraki, Japan) and housed in the animal facility at 22–24 °C with a humidity of 30–40 % on a 12-h (on)/12-h (off) light cycle with a light phase (8:00–20:00 h). Laboratory rodent chow (Lab MR Stock) and distilled water were provided ad libitum, unless otherwise specified.

On day 15 of gestation, pregnant rats were administered vehicle, TCDD in vehicle, or TBDD in vehicle by gavage at a dose of 0, 200, or 800 ng/kg body weight (six dams in each exposed group). On the second day after birth, the pups were culled to obtain five males and five females per litter that received a similar dose of TCDD or TBDD through lactation. These groups of offspring were accordingly named Control, TCDD-200, TCDD-800, TBDD-200, and TBDD-800 groups. Pups were allowed to be fed through lactation until postnatal day (PND) 21. The day of eye opening was examined and represented as the mean on a litter basis. Gross anatomical observations were made on PND 21 for one male offspring from each dam (six rats per each dose group).

At week 10, a male animal was randomly selected from each litter and housed individually. A total of 18 rats (six rats per each dose group) were used for the behavioral test, and each animal was allowed to have 20 g of rat chow per day because this regimen has been shown to maintain the body weight of rats to 80–85 % of their body weight from when the restriction was initiated. To study the possible effects of TCDD or TBDD effects on body weight, another male animal was randomly selected from each litter, and a total of 25 animals (five rats per each dose group) were used to monitor body weight under free-feeding conditions once a week.

The experimental protocols of the animal experiments of this study were approved by the Animal Care and Use Committee of the Graduate School of Medicine of the University of Tokyo, and the animals were treated humanely throughout this study.

### Behavioral apparatus and habituation

**Fig. 1:**
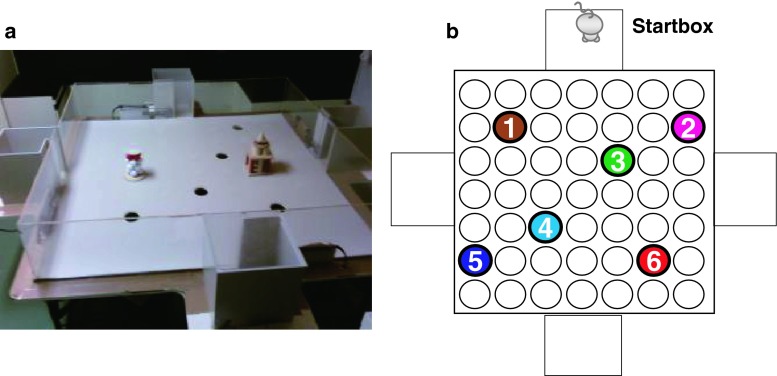
**a** The event arena is composed of a test arena [1,600 (w) × 1,600 (d) × 300 (h) mm] with a start box [250 (w) × 250 (d) × 300 (h) mm] at the center of each sidewall. **b** Arrangement of six flavor-location paired associations (F-L pairs) in the event arena. At each location (*1–6*), there was a well in which a specific-flavored rat chow (as described in the following parentheses) was concealed under sand.* 1* F1-L1 (chocolate);* 2* F2-L2 (cherry);* 3* F3-L3 (anise);* 4* F4-L4 (bacon);* 5* F5-L5 (coconut); and* 6* F6-L6 (strawberry). Rats were presented with flavored lab chow in the start box as a cue, and they were allowed to recall the spatial location with which it was associated and to go into the arena to search for the rat chow with the identical flavor (Tse et al. 2007)

The details of behavioral test of paired-associate learning for rats using the event arena apparatus have been described previously (Tse et al. 2007, 2011). In the event arena, sand wells can be placed at any location among a total of 49 locations on a 7 × 7 grid (Fig. 1a), in which rats have to dig through sand to obtain reward pellets as a reward.

In habituation of the present study, rats were trained to get used to the event arena apparatus and the foraging procedure in the event arena as follows: (1) eat a 190 mg flavored pellet in the start box, (2) enter and explore the event arena, (3) find a sand well and dig up the 190 mg pellet as a reward, and (4) carry the reward pellet back to the start box and eat it. Each rat experienced this procedure (=trial) six times a day. The location of the sand well and the start box varied in order to have rats thoroughly explore the event arena.

During habituation, the latency to enter the event arena from the start box was used as an index of anxiety-like behavior and was determined as time for rats to spend in the start box at the first training of each day.

The habituation was repeated up to day 30 when all the rats seemed to have thoroughly habituated themselves to the environment and have acquired the foraging behavior. As described in the “Results” section, both the TCDD- and TBDD-exposed groups showed anxiety-like behavior during habituation.

### Training of the paired association

The protocol of the present paired-associate learning test was identical to the previous report (Tse et al. 2007). In this test, a total of six sand wells were set at a fixed position in the event arena. Rats were required to find a sand well that contains three reward pellets (correct sand well, hereafter), while the other five sand wells contained no reward (incorrect sand wells, hereafter) in a trial. The 190 mg pellets flavored with one of the six flavors (i.e., chocolate, cherry, anise, bacon, coconut, and strawberry) were used as a cue and a reward for this test. In one trial, rats were given a pellet with a particular flavor in the start box (cue pellet, hereafter) and then allowed to enter the event arena to reach a correct sand well, the location of which was uniquely associated with the flavor of the cue pellet. All the sand wells were filled with sand mixed with finely crushed pellets with all the six flavors to mask the scent of the hidden reward. Therefore, rats had to learn the unique six flavor-location pairs to effectively find the correct sand well, obtain reward (correct choice, hereafter), and avoid digging the sand wells that contained no reward before making a correct choice (incorrect choice, hereafter) (Fig. 1b). When rats dug the correct sand well, rats were allowed to retrieve one of the three buried pellets at a time, and they brought one back to the start box to eat it. A trial was completed by repeating this foraging procedure three times for the same flavor-location pairs. Each daily session comprised a total of six trials for each of the six flavor-location pairs with an approximately 1-h interval between each trial. The location of the start box was switched every session. In order to evaluate learning achievement, performance index was calculated from the number of incorrect choice in the first foraging in each trial of the session as follows: 100–100*(the number of incorrect choice/5). Since there were five incorrect sand wells, the chance value of the number of incorrect choice and its performance index for each foraging procedure were 2.5 and 50, respectively.

In order to ensure that the rats’ foraging behavior was guided by paired-associate memory rather than other unintended cues such as scent that might have emanated from reward pellets hidden in a correct sand well, non-cued trials were conducted on session 46. In this session, a cue pellet was not presented to the rats in the start box in order to prevent them from foraging for food with the aid of paired-associate memory. If the rats showed a performance that was better than chance value in this session, it could be considered that the rats were guided by other unintended cues. In session 47, regular cued trials were conducted again.

### Index of the formation of simple memory

Rats are known to have a strong preference to dig the previous correct sand well during the several sessions from the beginning of the paired-associate learning test using the event arena (Tse et al. 2007). At the early phase of this test, such a behavior is considered as evidence that rats forage for food with the aid of  their simple episodic-like (or working) memory of the lastly rewarded event, but not that of paired-associate memory. Thus, we evaluated the simple memory in the rats using the following three indices. The index of “memory in seconds”: a probability to dig first the same correct sand well as the initial pellet foraging at the second pellet foraging through all the six trials in session 2. This index was based on the hypothesis that rats dig first the sand well from which they obtained a reward during the preceding few seconds within the same trial, if they remembered it. (2) The index of “memory in hours”: a probability to dig first the sand well that was correct in the previous trial at the initial foraging in each trial through the trials 2–6 in session 2. This index was based on the hypothesis that rats dig first the sand well from which they obtained a reward in the previous trial conducted an hour ago, if they remembered it. (3) The index of “memory in days”: a probability to dig first the correct sand well in the last, or sixth, trial of the previous day (session) in the initial foraging in trial 1 through sessions 2–7. This index was based on the hypothesis that rats dig first the sand well at the initial foraging of the next day, from which they obtained a reward in the last, or sixth, trial of the previous day, if they remembered it.

### Statistical analyses

All statistical analyses were conducted with an SPSS 15.0 (SPSS Japan Inc., Tokyo, Japan). A difference was considered significant with* p* values less than 0.05, unless otherwise specified. Data on the body weight of the dams or pups and the number of pups per dam were analyzed with two-way analysis of variance (ANOVA) with repeated measurements and Dunnett’s post hoc test. Changes in performance indices with sessions (days) were analyzed by repeated measurements with a Greenhouse-Geisser correction, including degrees of freedom. Other dose-response data were analyzed using one-way ANOVA or two-way ANOVA, which was followed by Dunnett’s post hoc test.

## Results

### General health status of TCDD- or TBDD-exposed rat offspring

TCDD administration on gestational day 15 at an oral dose of 200 or 800 ng/kg did not affect maternal body weight gain during gestation. All of the dams had a gestational period of 21 days, and they delivered 14–17 pups per litter without a significant difference in the number of pups among the Control, TCDD-200, and TCDD-800 groups.

No significant differences in body weight were observed among these groups during the period at least until PND 91, but the body weight of the TCDD-800 group was 10–15 % lower than that of the Control group during the period from PND 98 until PND154 (Table 1), despite the fact that the dose used in this study did not induce anatomical abnormalities (Suppl. Table 1) and the day of eye opening (Suppl. Table 2) that has been reported to be a sensitive marker in TCDD-exposed animals (Theobald and Peterson 1997). Besides, the dose was far below the one that has been shown to develop wasting syndrome, which leads to death within a few weeks post-administration (Rozman 1984; Seefeld et al. 1984). However, the body weight in the TCDD-200 group was similar to that of the Control group throughout the test. The TBDD-800 group seemed to decrease in body weight but missed a statistical significance. The body weight in the TBDD-200 group was similar to that of the Control group throughout the test (Table 1).

**Table 1:** Body weight gain (gram) after in utero and lactational exposure to TCDD or TBDD

	Control	200 ng TCDD/kg	800 ng TCDD/kg	200 ng TBDD/kg	800 ng TBDD/kg
PND 56	316 ± 9.8	310 ± 9.2	299 ± 10.9	310 ± 6.2	304 ± 6.1
PND 70	380 ± 15.7	368 ± 12.5	355 ± 15.0	380 ± 6.6	367 ± 5.9
PND 84	440 ± 21.3	421 ± 12.1	384 ± 17.3	406 ± 17.3	412 ± 7.1
PND 98	490 ± 22.5	464 ± 13.1	424 ± 16.4*	470 ± 11.4	452 ± 7.3
PND 112	522 ± 23.8	497 ± 13.6	449 ± 18.8*	500 ± 8.8	484 ± 11.3
PND 126	559 ± 26.4	538 ± 11.3	476 ± 18.2**	529 ± 10.5	515 ± 11.1
PND 140	587 ± 27.6	579 ± 5.8	498 ± 21.4***	561 ± 13.7	537 ± 11.7
PND 154	610 ± 28.2	599 ± 9.4	522 ± 21.8***	583 ± 12.2	560 ± 16.0

Mean ± SE for 5 animals. * *p* < 0.05, ** *p* < 0.01, and *** *p* < 0.001 versus control by one-way ANOVA with Dunnet’s post hoc test

### Anxiety-like behavior in TCDD- or TBDD-exposed rat offspring

On the first day of habituation, the time spent by the Control group in the start box before they entered the arena was approximately 5 min (on an average), and it rapidly decreased to approximately 30 s in the first 3 days (data not shown). The latency was reduced to 10 s on the fifth day. This result was consistent with the results of a previous report (Tse et al. 2007). However, the TCDD-200 group showed a significantly longer latency than the Control group from days 1–15 of habituation (Table 2). No significant differences in the latencies were observed after day 20, and the habituation was extended for 30 days. Although prolonged latency has been considered to reflect anxiety status (Ennaceur 2012), the completion of habituation assures that evaluations of the prospective behavioral test results can be interpreted to reflect learning and memory function independent from emotion. In addition, we confirmed that there was no extension of latency during the following memory test.

**Table 2:** Latency (second) for rats to enter the event arena during habituation

	Control	TCDD (ng/kg)		TBDD (ng/kg)	
		200	800	200	800
Day 1	289.2 ± 70.3	394.6 ± 60.0*	329.5 ± 50.1	466.4 ± 85.5*	313.2 ± 95.9
Day 5	10.5 ± 1.73	28.2 ± 10.0*	17.0 ± 4.3	67.0 ± 20.0*	26.5 ± 9.3
Day 10	6.7 ± 1.9	19.3 ± 4.7*	6.3 ± 1.5	21.6 ± 2.1*	9.8 ± 2.1
Day 15	9.8 ± 4.4	15.3 ± 2.2*	8.3 ± 1.6	21.4 ± 10.9	13.0 ± 3.2
Day 20	3.3 ± 0.9	6.0 ± 1.1	4.0 ± 0.4	2.5 ± 0.4	2.6 ± 0.4

Mean ± SE for 5 animals

* p < 0.05 versus control by one-way ANOVA with Dunnet’s post hoc test

In utero and lactational exposure to TBDD at 200 or 800 ng/kg resulted in essentially the same results as those observed in the TCDD-exposed groups. Although no difference in latency was observed between the TBDD-800 and Control groups, latency was significantly extended in the TBDD-200 group compared with the Control group (Table 2).

### Paired-associate learning in in utero and lactational exposure to TCDD and TBDD

**Fig. 2:**
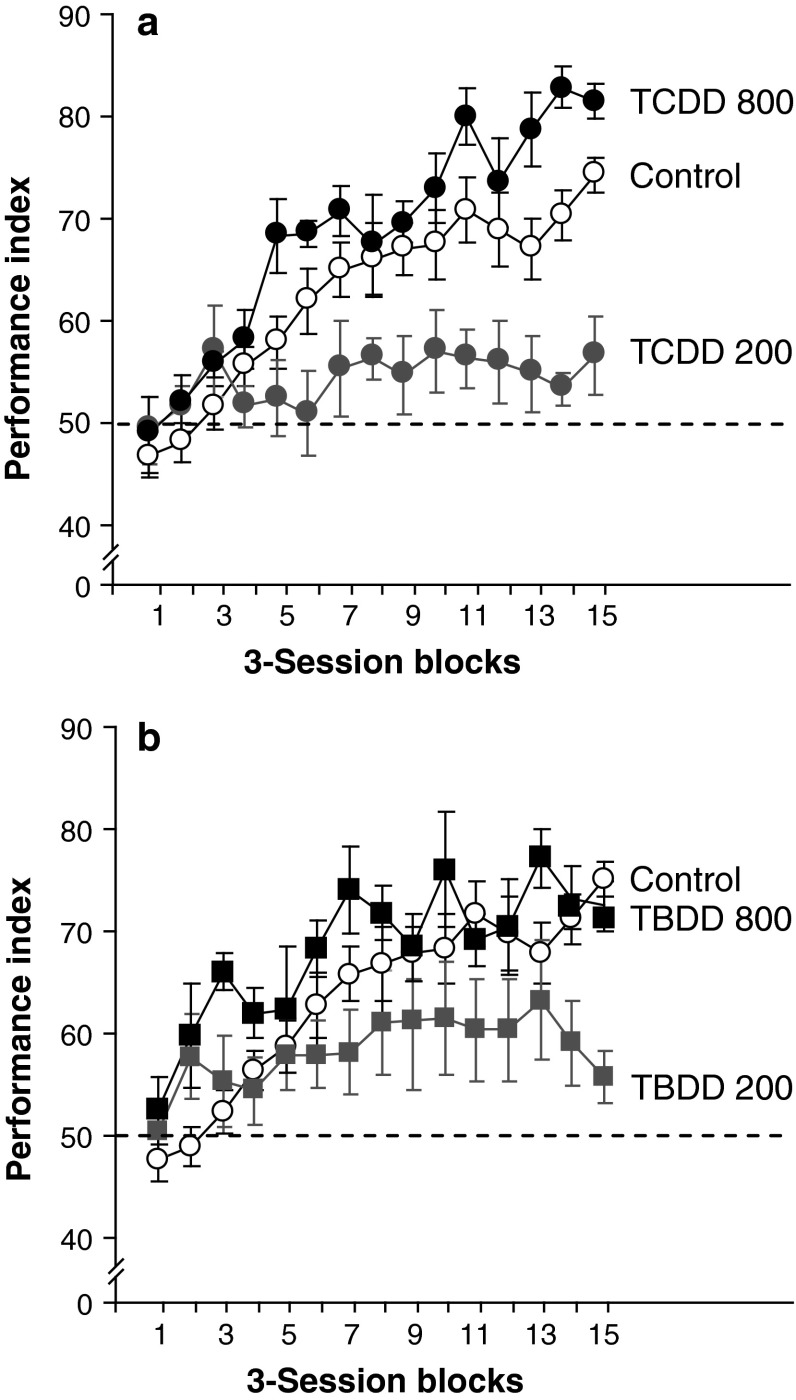
Acquisition of paired-associate memory in the behavioral test. For readability, the performance index in the 3-session block, which comprised data from each animal per session for three consecutive sessions, is shown. Control, offspring born to dams that were administered corn oil; TCDD 200 and TCDD 800, offspring born to dams that were administered TCDD at an oral dose of 200 and 800 ng/kg, respectively; TBDD 200 and TBDD 800, offspring born to dams that were administered TBDD at an oral dose of 200 and 800 ng/kg, respectively. The Control group was commonly compared with the TCDD- and TBDD-exposed groups. **a** The performance indices of the Control and TCDD-800 groups increased with the sessions and were significantly higher than the chance value [Control, *p* < 0.001, *F*(1,14) = 14.6; TCDD-800, *p* < 0.001, *F*(1,14) = 15.1], whereas the performance index of the TCDD-200 group did not differ from the chance value [TCDD-200, *p* = 0.87, *F*(1,14) = 0.58]. The performance index of the TCDD-200 group was significantly lower than that of the Control group [*p* < 0.05, analysis of variance with Dunnett’s post hoc test]. **b** The performance index of the TBDD-exposed rat offspring showed a similar trend as that observed in TCDD-exposed rats. The performance index of the TBDD-800 group was significantly higher than the chance value [*p* < 0.001, *F*(1,14) = 4.58], whereas no significant difference was observed for the TBDD-200 group [*p* = 0.35, *F*(1,14) = 1.14]

The performance index of the Control group was at chance level (performance index = 50) in the beginning of the behavioral test and gradually increased up to 75 during the sessions. It was significantly increased above chance value from session 26 (3-session block 8) to session 45 (3-session block 15) (Fig. 2). However, no training effects that increased the performance index were observed in the TCDD-200 group, and the performance index of this group was not significantly different from the chance level throughout the sessions. However, it was significantly lower than the Control group at session 26 (3-session block 8) and thereafter (Fig. 2), suggesting a perturbation in the formation of paired-associate memory. However, the performance index of the TCDD-800 group increased with the sessions, and it was similar to that of the Control group, without a sign of any adverse effects of TCDD (Fig. 2).

The in utero and lactational exposure to TBDD resulted in essentially the same dose-response patterns as those observed with TCDD exposure: The performance index of the TBDD-200 group was significantly lower than that of the Control group, although the difference between them was not as conspicuous as that observed between the Control and TCDD-200 groups. Moreover, the TBDD-800 group showed a performance index level similar to that of the Control group.

**Fig. 3:**
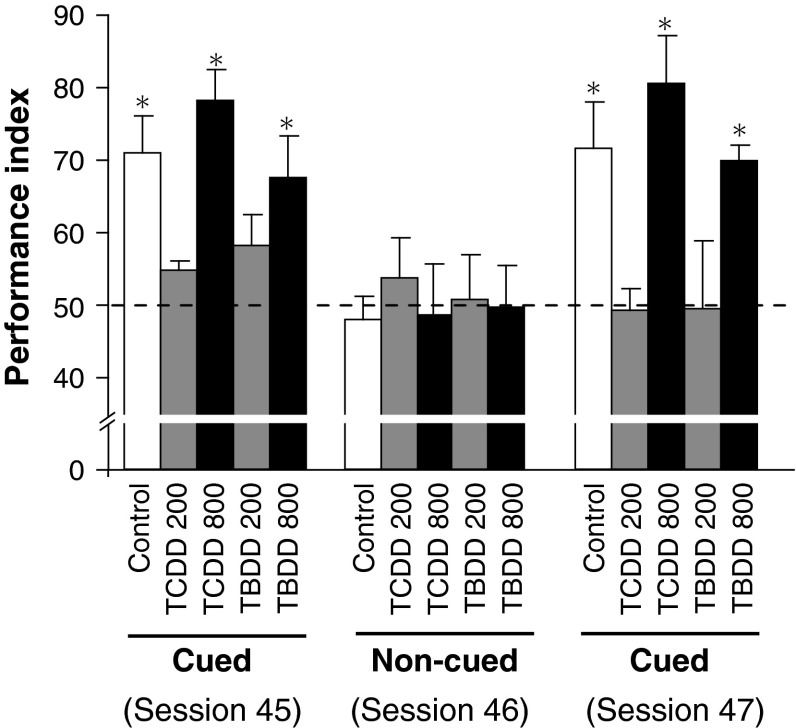
Verification of paired-associate learning by non-cued trials. The non-cued session (session 46; six non-cued trials), followed by the cued session (session 47; regular session), was assessed in the last part of the experiment. In the non-cued condition, a flavored diet pellet as a cue was not set in the start box, and a reward-flavored diet pellet was set in a sand well. * *p* < 0.05 versus  chance value

Non-cued probe trials were conducted in session 46 where a cue pellet was not presented in the start box. As a result, in the non-cued trials, the performance indices of all the groups did not significantly differ from the chance value. On the other hand, in sessions before and after the non-cued session (i.e., session 45 and 47), where a cue pellet was presented in the start box, the performance indices of the Control, TCDD-800, and TBDD-800 groups were significantly higher than the chance value consistently (Fig. 3). Therefore, it was ensured that the differences of the performance indices observed among the groups in this study were attributable to paired-associate learning ability.

### Evaluation of simple memory indices

**Fig. 4:**
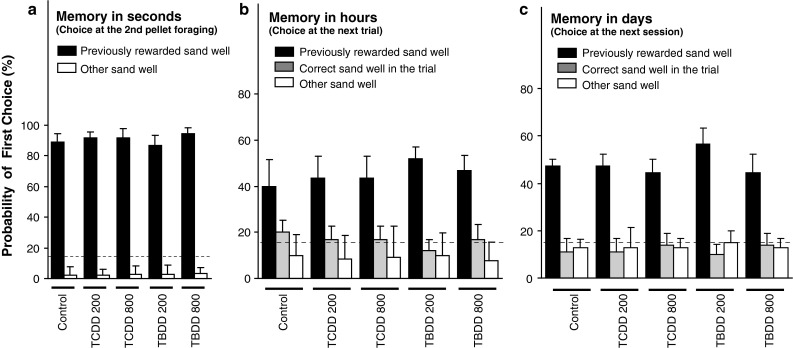
Simple memory formation. Between-group difference in simple memory performance was assessed with the following three indices of simple memory: **a** “memory in seconds”, **b** “memory in hours”, and **c** “memory in days”. *Black bars* correspond to the indices that represent the probability to choose the previously rewarded sand well as the first choice (but see “Materials and methods” for each detailed definition). *Gray bars* indicate the probability to choose the correct sand well in the session which was different from the previously rewarded one. *White bars* indicate the probability to choose other sand wells as the first choice. *Dotted line* indicates a chance level (16.7 %)

The three simple memory indices defined in “Materials and methods” did not significantly differ among all the groups (Fig. 4). This result indicated that neither TCDD-200 nor TBDD-200 group affected such simple forms of memory of the previously rewarded location that was available within seconds, hours, and days.

## Discussion

### Impairment of paired-associate learning by maternal dioxin exposure

One of the most significant findings of this study was that paired-associate learning was perturbed in rat offspring born to dams that were given a low TCDD dose (=200 ng/kg). Excessive exposure to a variety of chemicals in general and occupational settings is a major threat to human health. In particular, the developmental brain has been established to be extremely vulnerable to such chemical exposure (World Health Organization 1998). Epidemiological and laboratory studies have demonstrated that neurobehavioral abnormalities, which cannot be necessarily diagnosed on an individual basis, could be induced in offspring by maternal exposure to chemicals, including dioxins. A wide repertoire of behavioral and cognitive tests that are used to examine learning, memory, and emotion has been applied to rat offspring that were exposed to TCDD in utero and through lactation. A low TCDD dose that did not overtly affect the dams disrupted the learning behavior of the offspring when they were challenged by behavioral tests (Hojo et al. 2002; Markowski et al. 2002). Thus, we concluded that maternal exposure to a low TCDD dose could affect the offspring, but it was difficult to delineate the toxicity mechanisms based on the fact that these effects were often differentially observed depending on the sex, dose, animal species, and test methods. Application of this behavioral test to relatively low TCDD- and TBDD-exposed groups showed that it was robust enough to produce data with high reproducibility over at least 2 months. The validity of this test was shown by a series of tests involving cued and non-cued sessions (Fig. 3). That is, the performance indices of the low-dose (TCDD-200 and TBDD-200) groups showed that the perturbations in paired-associate learning were indifferent to the presence of the cue, and those of the Control and high-dose (TCDD-800 and TBDD-800) groups decreased to the chance value in the non-cued session, but recovered above the chance value in the subsequent cued sessions. These results clearly demonstrated that the performances of these three groups were mediated by a cue flavor at the start box rather than other uncontrolled cues, and that TCDD and TBDD at low doses perturbed paired-associate learning.

Paired-associate learning typically consists of a study phase of pairs of items, such as a flavor of food and its spatial location, as in this study, which is followed by a test of cued recall, and the hippocampal glutamate *N*-methyl-d-aspartate (NMDA) receptors have been reported to be crucial for the formation of a paired association in the event arena in rats (Day et al. 2003). This behavioral test can assess not only the ability of rats to memorize each of six paired associates, but also their ability to learn a set of maps (Tse et al. 2007). Because schema-based learning has been found to be dependent on neocortical function (Tse et al. 2011), the present results suggested that TCDD and TBDD at a low dose can perturb neocortical function. This speculation was supported by previous observations that maternal exposure to TCDD inhibited activity-dependent gene expression in the frontal cortex (Kakeyama et al. 2003) and that the gene expression of the NMDA receptor subunit in the neocortex was affected by TCDD (Hood et al. 2006; Kakeyama et al. 2001; Nayyar et al. 2003).

Next, anxiety-like behavior was induced in rat offspring upon maternal exposure to TCDD (Table 2). Because the training for the behavioral test started after habituation and because the activity of rats in terms of the distance of movement and velocity did not differ among the groups (data not shown), it was reasonable to speculate that dioxin-induced disruption of learning ability, but not that of emotional function, was responsible for the significant reduction in the paired association.

### Comparable toxicity of TCDD and TBDD

Another significant observation was that both TCDD and TBDD similarly disrupted higher brain function. The biological and toxic features of polybrominated dibenzodioxins and dibenzofurans have been considered to be similar to those of their corresponding chlorinated congeners (World Health Organization 1998). A recent review article (van den Berg et al. 2013) has described that brominated congeners have a relative potency that is similar to that of their corresponding chlorinated congeners in a variety of experimental systems, including memory and emotional functions, in vivo (Haijima et al. 2010). In this particular study, maternal exposure to either TCDD or TBDD inhibited the fear memory function in mice (3.0 μg/kg body weight on gestational day 12.5 to the dam of C57BL/6 mice), indicating that the potency of TBDD was similar to that of TCDD, at least for developmental neurotoxicity. Although studies on the comparative toxicities of TBDD and TCDD in vivo are limited, the present results provided another example of the similar magnitude of the potencies of chlorinated and brominated congeners of dioxins in anxiety-like behavior (Table 2) and disruption of paired-associate learning (Figs. 2, 3) at the low (200 ng/kg) dose and in body weight loss (Table 1) at the high (800 ng/kg) dose. Observations in in vivo studies can differ each other  depending on what kinds of endpoints were used. For example, TBDD has been reported to be half effective on the induction of cleft palate and nearly three times as potent as TCDD on immune function (Birnbaum et al. 2003). Although most of the in vivo studies that have been conducted had the insufficient numbers of dose points, it can be generally concluded that the relative potency of 2,3,7,8-substituted polybrominated dibenzodioxin and polybrominated dibenzofuran is comparable with that of their corresponding chlorinated congeners in mammalian systems (van den Berg et al. 2013).

### Nonmonotonic *U* shaped dose-response phenomenon

The last, but not the least, significant observation was the nonmonotonic *U* shaped dose-response that was elicited by TCDD or TBDD in the present study. As described in the above section, the low-dioxin-exposed (TCDD-200 and TBDD-200) groups had anxiety-like behavior, unlike the high-dioxin-exposed (TCDD-800 and TBDD-800) groups and the Control group. Findings of such a nonmonotonic *U* shaped dose–response curve in behavioral animal studies have not been rare, but have often been reported by many laboratories (Vandenberg et al. 2012). Male offspring that were born to Sprague-Dawley rats that were exposed to a total dose of 0.7 μg TCDD/kg showed decreased working memory, whereas those that were exposed to a total dose of 1.4 μg TCDD/kg exhibited behavior that was similar to that of the controls (Seo et al. 2000). In another study (Markowski et al. 2002), male and female offspring that were born to Holtzman rats that were exposed to 0.18 μg TCDD/kg were significantly less accurate in cued delayed alternation procedures that were used to examine operant behavior compared with the other two groups (control and 0.54 μg TCDD/kg). Previous observations have also indicated that there is a low-dose-specific effect of maternal exposure to TCDD (Hojo et al. 2002). In male and female offspring that were born to Long-Evans hooded rats that were exposed to TCDD, the animals of the medium-dosed group (200 ng/kg) had an increased tendency in behavioral performance compared with the other three groups (0, 50, or 800 ng TCDD/kg) (Hojo et al. 2008). Nonmonotonic *U* shaped dose-response behavior was observed both in rat offspring, as described above, and mouse offspring. Mice born to dams that were exposed to TCDD (0, 0.6, or 3.0 μg TCDD/kg) were housed together in a behavioral test apparatus and examined for behavioral flexibility, perseverative behavior, and competitive dominance when they reached adulthood. The low-dosed (0.6 μg TCDD/kg) group of animals was significantly different from the other two groups for these three endpoints (Endo et al. 2012). There is no doubt that the dose-response curves of endocrine disrupting chemicals, as well as natural hormones, conform to the nonmonotonic *U* shaped dose-response phenomenon (Vandenberg et al. 2012). It can be speculated that a low dose of TCDD and the natural ligand may exert a rigorous physiological response until excess amounts of their receptor in the cytoplasm, named arylhydrocarbon receptor, were fully saturated. At a higher dose, TCDD could competitively inhibit with endogenous ligand binding, thus reducing the effect (Markowski et al. 2002). This speculation is supported by spare receptor theory, which has been widely accepted in pharmacology. Besides the receptor-mediated mechanism, the altered expression of a certain gene product may explain such a nonmonotonic dose-response relationship. For example, mice with a targeted CREB hypomorphic mutation were reported to show profound deficits in hippocampus-dependent task performance (Bourtchuladze et al. 1994), while the mice lacking all the CREB isoform genes in hippocampus were less detrimental (Balschun et al. 2003). These observations can be explained by compensatory upregulation of CREM and/or other transcription factors that might rescue the loss of CREB functions (Balschun et al. 2003). However, the underlying molecular mechanism is still elusive and warrants future studies. Taken together, the present results showed that the maternal exposure to dioxins, either TCDD or TBDD, at a low dose perturbed higher brain function of offspring in a low-dose-specific manner.

Paired-associate learning is a fundamental constituent of the intellectuality of humans that allows for language acquisition and a vast store of knowledge. In this study, we robustly revealed that paired-associate learning was affected by the developmental exposure to a low dose of environmental chemicals. Given the possible global effects of environmental chemicals on the intellectual development of children, the present animal test should be added to the repertoire of test batteries of developmental neurotoxicity that are currently used.

## Electronic supplementary material

Below is the link to the electronic supplementary material.

Supplementary material 1 (DOCX 17 kb)

## Abbreviations

ANOVA: Analysis of variance
TBDD: 2,3,7,8-Tetrabromodibenzo-*p*-dioxin
TCDD: 2,3,7,8-Tetrachlorodibenzo-*p*-dioxin
