# Supplementary material for: Disruption of paired-associate learning in rat offspring perinatally exposed to dioxins
Source: Arch Toxicol. 2013 Nov 29;88(3):789–98. doi: 10.1007/s00204-013-1161-y (PMC3927058; doi:10.1007/s00204-013-1161-y)
Supplement: Supplementary file 1 — Supplementary material 1 (DOCX 17 kb) [file 204_2013_1161_MOESM1_ESM.docx]

**Supplementary Document:**

**Disruption of paired-associate learning in rat offspring perinatally exposed to dioxins**

Masaki Kakeyama^1,2^, Toshihiro Endo^1^, Yan Zhang^1^, Wataru Miyazaki^1,3^ and Chiharu Tohyama^1^

^1^Laboratory of Environmental Health Sciences, Center for Disease Biology and Integrative Medicine, Graduate School of Medicine, The University of Tokyo, 7-3-1 Bunkyo-ku, Tokyo 113-0033, Japan.

^2^Department of Neurobiology and Behavior, Graduate School of Biomedical Sciences, Nagasaki University, 1-7-1 Sakamoto, Nagasaki, 852-8501, Japan

^3^Department of Public Health, Faculty of Life Sciences, Kumamoto University, 1-1-1 Honjyo, Kumamoto 860-8556, Japan

| Supplementary Table 1. Gross anatomical changes in offspring from TCDD- or TBDD- exposed dams^a^ | | | | | | | |
| --- | --- | --- | --- | --- | --- | --- | --- |
|  | |  | Control | 200 ng TCDD/kg | 800 ng TCDD/kg | 200 ng TBDD/kg | 800 ng TBDD.kg |
|  | |  |  |  |  |  |  |
|  | Number of Dams | | 6 | 6 | 6 | 6 | 6 |
|  | Number of offspring | | 6 | 6 | 6 | 6 | 6 |
| No abnormality | | | 6 | 6 | 5 | 6 | 6 |
| Kidney | | |  |  |  |  |  |
| Dilatation, pelvis | | | 0 | 0 | 0 | 0 | 0 |
| Hydronephrosis | | | 0 | 0 | 0 | 0 | 0 |
| Testis | | |  |  |  |  |  |
| Small | | | 0 | 0 | 1 | 0 | 0 |
| Enlargement | | | 0 | 0 | 0 | 0 | 0 |
| Epididymis | | |  |  |  |  |  |
| Small | | | 0 | 0 | 0 | 0 | 0 |
| Genitalia | | |  |  |  |  |  |
| Cleft prepuce | | |  |  |  |  |  |
| Abdominal cavity | | |  |  |  |  |  |
| Spleen and peritoneum Adhesion, | | | 0 | 0 | 0 | 0 | 1 |
| Duodenum and uterus Adhesion, | | | 0 | 0 | 0 | 0 | 0 |
| a. One male pup was randomly selected from each dam and was subjected to gross anatomical observation. | | | | | | | |

| Supplementary Table 2. The day of eye opening after birth in male offspring^a^ | | | | | |
| --- | --- | --- | --- | --- | --- |
|  | Control | 200 ng  TCDD/kg | 800 ng  TCDD/kg | 200 ng  TBDD/kg | 800 ng  TBDD/kg |
| Male | 15.3 ± 0.13 | 14.9 ± 0.13 | 15.0 ± 0.10 | 15.2 ± 0.12 | 15.0 ± 0.13 |
| 1. Mean ± SE for 5 animals. The mean of the day of eye opening was calculated on a litter basis after averaging the day of eye opening of offspring from each dam. | | | | | |
